# The role of the anterior, mediodorsal, and parafascicular thalamus in instrumental conditioning

**DOI:** 10.3389/fnsys.2013.00051

**Published:** 2013-10-09

**Authors:** Laura A. Bradfield, Genevra Hart, Bernard W. Balleine

**Affiliations:** Behavioral Neuroscience Laboratory, Brain and Mind Research Institute, University of SydneySydney, NSW, Australia

**Keywords:** anterior thalamic nuclei, mediodorsal thalamic nucleus, parafascicular thalamic nuclei, corticothalamic disconnection, prelimbic cortex, instrumental conditioning

## Abstract

The traditional animal model of instrumental behavior has focused almost exclusively on structures within the cortico-striatal network and ignored the contributions of various thalamic nuclei despite large and specific connections with each of these structures. One possible reason for this is that the thalamus has been conventionally viewed as a mediator of general processes, such as attention, arousal and movement, that are not easily separated from more cognitive aspects of instrumental behavior. Recent research has, however, begun to separate these roles. Here we review the role of three thalamic nuclei in instrumental conditioning: the anterior thalamic nuclei (ANT), the mediodorsal (MD), and parafascicular thalamic nuclei (PF). Early research suggested that ANT might regulate aspects of instrumental behavior but, on review, we suggest that the types of tasks used in these studies were more likely to recruit Pavlovian processes. Indeed lesions of ANT have been found to have no effect on performance in instrumental free-operant tasks. By contrast the mediodorsal thalamus (MD) has been found to play a specific and important role in the acquisition of goal-directed action. We propose this role is related to its connections with prelimbic cortex (PL) and present new data that directly implicates this circuit in the acquisition of goal-directed actions. Finally we review evidence suggesting the PF, although not critical for the acquisition or performance of instrumental actions, plays a specific role in regulating action flexibility.

## Introduction

The thalamus has been traditionally viewed as a sensory relay center, forming the interface between the sensory cortices and subcortical structures responsible for the execution of actions. In performing this role, several thalamic nuclei have been implicated in general processes such as arousal, attention, and voluntary movement. However, research within the last three decades has begun to focus more specifically on the role of the thalamus in instrumental conditioning. This has been driven, at least in part, by anatomical evidence that many thalamic nuclei have large and specific connections with the prefrontal cortex and dorsal striatum that now have well-established roles in the regulation of instrumental learning and performance.

In this review, we examine studies that have incorporated behavioral tasks in conjunction with various neural manipulations to assess the role of individual thalamic nuclei in instrumental learning and behavior. In particular we will consider the role of three thalamic nuclei: the ANT, mediodorsal (MD) and parafascicular thalamic nuclei (PF), the latter often referred to, in humans and primates, as the centromedian-parafascicular complex. In evaluating the evidence that any neural structure plays a role in some specific function, however, it is necessary to carefully evaluate, not only the anatomical but also the functional evidence. In the case of instrumental conditioning that requires evaluating the behavioral evidence that specific anatomical manipulations are influencing instrumental actions and not other forms of conditioned behavior and, in the current context, the chief alternative to instrumental action is, of course, the Pavlovian conditioned response.

Pavlovian conditioning occurs through pairings of an initially neutral stimulus, the conditional stimulus (CS), and a biologically relevant unconditional stimulus (US). Across repeated pairings, the CS comes to elicit a specific set of conditioned responses (CRs) indicative of the animal’s expectancy of the impending US. By contrast, instrumental conditioning involves the animal learning to perform (or withhold) an action dependent on its consequences. Although the distinction between them may appear clear enough, in reality it is complicated by the fact that instrumental conditioning often takes place in the presence of stimuli any of which could form Pavlovian stimulus-outcome (S-O) relations. Under some circumstances, therefore, it might be difficult to separate whether it is the instrumental contingency or the Pavlovian S-O contingency that is guiding behavior.

There are two criteria that distinguish instrumental actions from Pavlovian CRs (cf. Dickinson and Balleine, 1994). Specifically, whereas an agent should be able to withhold and/or flexibly alter (e.g., reverse) the direction of an instrumental response to obtain an outcome (cf. Dickinson, 1994), the same is not true of Pavlovian CRs. Thus, whereas it is clear that rats are capable of withholding a lever press response to receive a pellet outcome (Davis and Bitterman, 1971) and, further, that this response can be bidirectional; a lever can be pushed either up or down to gain a reward (Bolles et al., 1980; Dickinson et al., 1996), Pavlovian CRs are not open to such adjustment (e.g., Hershberger, 1986), nor can the response be withheld during the stimulus to gain the reward (Sheffield, 1965; Williams and Williams, 1969; Holland, 1979).

These examples demonstrate that Pavlovian CRs are controlled by S-O relations. In contrast, evidence suggests that, in instrumental conditioning, development of the instrumental action can be controlled by two other distinct forms of learning process. Considerable evidence suggests that instrumental actions can be goal-directed and controlled by the encoding of specific response-outcome (R-O) relationships. Much of this evidence has been provided by outcome devaluation studies (e.g., Adams and Dickinson, 1981; Colwill and Rescorla, 1985; Dickinson and Balleine, 1994). In such studies animals are trained to perform a response for a particular outcome, the value of which is subsequently reduced by feeding it to satiety or repeatedly pairing it with lithium chloride to induce illness. The animal is then tested for its propensity to make that response under extinction (i.e., in the absence of feedback from outcome delivery). If animals subsequently shows reduced performance of the response previously paired with the now devalued outcome this can be taken as evidence that it is goal-directed (Dickinson and Balleine, 1994) because it is governed by both: (1) a representation of the outcome as a “goal” and (2) a representation of the contingency between performance of the action and access to the outcome. The absence of feedback on test ensures that the second criterion is met because the animal can only rely on its prior knowledge of the R-O contingency to show the requisite reduction in performance.

Importantly, continuing performance on a lever after devaluation, as has been reported after extended training, demonstrates that performance is sometimes not guided by its relation with the outcome. Such demonstrations (Adams, 1982; Dickinson et al., 1995; Yin et al., 2004, 2006; Lingawi and Balleine, 2012) have been argued to reflect the behavioral development of habits. Habits are not guided by the R-O relation but, rather, reflect the role of the outcome as a reinforcer, strengthening the relation between prevailing stimuli (S) such as the context and the response (R). Behavioral and neurological evidence (Dickinson et al., 1995; Yin et al., 2004, 2005a,b) suggests that S-R and R-O relations are not mutually exclusive and develop in parallel with the influence over performance shifting across the course of training. Although the behavioral and neural processes that control habitual actions are important and of increasing interest, in this review we will refer primarily to goal-directed instrumental action whose performance is under the control of the R-O relation.

Finally, although the learning processes controlling the Pavlovian CR are distinct from those controlling instrumental actions, the latter actions can be influenced by specific retrieval-related effects of Pavlovian stimuli, an effect demonstrated using the Pavlovian-instrumental-transfer (PIT) paradigm. In such procedures, Pavlovian S-O and instrumental R-O relations are trained separately, and the ability of the Pavlovian stimuli to modulate instrumental performance is measured in an extinction test. The typical finding is that, on test, stimulus presentations promote responding on the instrumental action that was paired with the same outcome during training. For example, Colwill and Rescorla (1988) showed that a tone that had been paired with pellets promoted the performance of instrumental actions that had also been paired with pellets, relative to actions that earned a different outcome during training. This specific PIT effect requires the ability of the animal to retrieve specific R-O relations based on the ability of the Pavlovian stimulus to evoke a representation of the outcome. As a consequence, this effect is often characterized in terms of the formation of an S-O → R process in which the stimulus based retrieval of a specific outcome causes the animal to retrieve its specific associated action (see Balleine and Ostlund, 2007; Balleine and O’Doherty, 2010, for discussion).

In the remainder of the paper, we examine the aforementioned thalamic structures and their role in instrumental conditioning, focussing specifically on their role in goal-directed actions. With regard to the issues above, therefore, we will attempt to focus on actions that have been shown to be acquired and maintained by their contingent relationship to, and the value of, their consequences, rather than by antecedent stimuli. Where relevant, therefore, we will point to issues of behavioral control affecting interpretation and that may require clarification in future studies.

## Anterior thalamic nuclei

**Figure 1:**
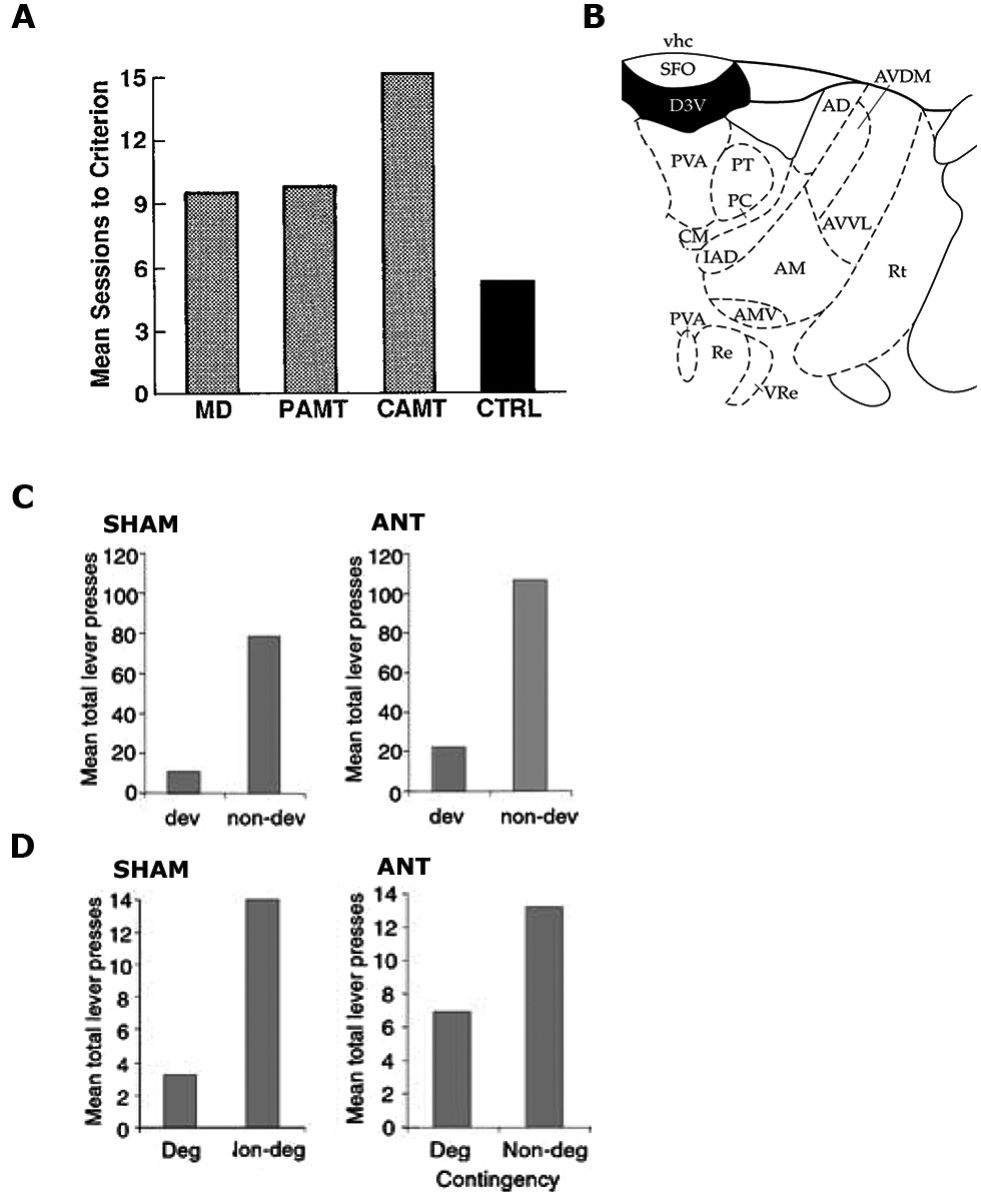
**(A)** Reproduced from Gabriel et al. (1989). Mean sessions to criterion responding for rabbits with mediodorsal thalamus (MD), partial medial dorsal and anterior thalamic (PAMT), combined medial dorsal and anterior thalamic (CAMT) and control (CTRL) lesions. **(B)** Reproduced from Paxinos and Watson (1998). Schematic showing ANT at A/P: −1.4 from Bregma. **(C,D)** Reproduced from Corbit et al. (2003), examines Sham and ANT-lesioned animals. **(C)** Mean total lever-press responses for the outcome devaluation test. **(D)** Mean total lever-press responses for the contingency degradation test.

Several studies spanning the late 1970s–early 2000s proposed that the anterior thalamic nuclei (ANT: see Figure 1B) play a role in the regulation of behavior in discrimination tasks involving instrumental responding. In particular, a series of experiments by Gabriel et al. (1977, 1983, 1989) found several lines of evidence to suggest ANT involvement in the learning that underlies performance in a series of avoidance and appetitive discrimination tasks in rabbits.

The earliest of these studies examined unit recordings from the anterior cingulate cortex (AC), the reciprocally connected ANT, or both, during an aversive avoidance task. In this task the presentation of a tone stimulus (S+) preceded the presentation of a footshock. Rabbits also received presentations of a different frequency tone stimulus (S−) that did not predict shock. This procedure was carried out in a running wheel and the consequence of the rabbit performing a wheel turn during the S+ presentation was avoidance of the footshock as well as termination of the S+. Similar responses during S− presentations also terminated the S−. Behaviorally, rabbits learnt this task relatively well, taking between 4–5 sessions on average to reach a criterion of 9–10 responses to the CS+ and 9–10 non-responses to the CS− (Gabriel et al., 1977).

The first examination of the AC-ANT pathway using this task was conducted by Gabriel and colleagues (Gabriel et al., 1977). In this study it was found that neuronal activity increased from baseline in both the AC and ANT in the 15–25 ms following stimulus onset and decreased from 35–75 ms, then increased again at 75 ms where it continued until 200 ms when recording ceased. This response was greater in magnitude to the S+ than the S− in the first 100 ms and, as a consequence, the authors proposed that these differential neural responses reflected discrimination learning and that this information was used to evoke a behavioral response to the S+ over the S−. This experiment was one of the first attempts to apply a psychological function to a thalamic region that could be separated from the regulation of some other general function. In particular, the authors claimed that arousal and body orientation could not have influenced the results because these states should have been the same prior to the onset of both the S+ and the S− such that any differential responding to each stimulus could only be elicited as a result of their differential relationships with the footshock.

A later study (Gabriel et al., 1989) demonstrated a causal role for the ANT in regulating the underlying learning in the discriminative avoidance task outlined above. Gabriel et al. (1983) had previously shown that bilateral ANT lesions eliminated excitatory responses to the S+ in the cingulate cortex and, as such, they hypothesized that lesioning the ANT might also affect behavior in this task. They found that rabbits with combined lesions of the MD and ANT were unable to reach criterion, whereas rabbits with only MD lesions did not differ from controls. Rabbits with MD lesions did show some impairment relative to controls when their percentage of correct responses to the S+ relative to S− was considered, but rabbits with combined ANT/MD lesions were more impaired than either group (see Figure 1A). Again, the authors claimed that these differences could not be attributed to deficits in the general processes of orienting or autonomic responses to the stimuli as these were intact in lesioned animals. Further, the aversive footshock was argued to be similarly effective in all of the rabbits. As a consequence, the authors attributed the impaired performance to a deficit in learning.

There have been several follow-up studies replicating and expanding on these earlier effects. A notable example is that of Smith et al. (2002) who used an appetitively motivated discrimination task to examine the role of ANT and MD in appetitive conditioning. For this task, a water reward was given after head extension and oral contact with a spout following a tone S+ presentation whereas no reward was given after the (alternate frequency) tone S−. Rabbits with limbic thalamic lesions (spanning ANT and MD) were severely impaired in their acquisition of the task, but did eventually reach criterion. Further, cingulate cortical neurons developed discriminative neuronal responses (S+ > S−) in controls but not lesioned rabbits. These results were interpreted as implicating the limbic thalamic-AC pathway in associative learning more generally, rather than aversive avoidance learning specifically.

These and other studies (e.g., Sparenborg and Gabriel, 1992; Gabriel et al., 1995) represent, therefore, a significant body of work implicating the ANT-AC pathway and to a lesser extent the MD, in discrimination learning. It should be emphasized that the authors did not claim a role for this pathway in the regulation of instrumental behavior *per se*, but rather referred to their discrimination task as requiring an instrumental response. However, none of these studies included a specific test of the bidirectionality or omission of these responses, so it remains open to question as to whether they were actually instrumental or subject to other, particularly Pavlovian, contingencies. The head extension response in particular (Smith et al., 2002) seems an unlikely candidate for an instrumental response as it comprises a food approach behavior, which cannot be withheld or flexibly performed to achieve a desired outcome. The wheel turn response, on the other hand, comprises a better candidate for an instrumental response as it has been shown to be sensitive to omission (Wilson et al., 1987). Although to our knowledge bidirectional performance of wheel turning has not been demonstrated, it is not unreasonable to think that if a rabbit can turn a wheel in one direction to avoid a shock it could turn it in the opposite direction for the same outcome.

What is not clear from these studies, however, is the type of relation governing performance in these particular tasks. Because footshock occurred only in the presence of the S+, it is possible that in spite of its potentially instrumental nature, wheel turn responding in the presence of the S+ simply constituted a conditional response governed by S-O relations. Indeed, if wheel-turning might be considered a form of escape, which is an unconditional response appropriate to footshock, then this response could even be seen to fulfil Pavlov’s (1927) criterion of stimulus substitution. Even if we do accept that there was an instrumental contingency between wheel turning and shock avoidance, the fact that performance of this response only led to the desired outcome (i.e., footshock avoidance) in the presence of the S+ creates the possibility that it was under Pavlovian control in a manner similar to that observed during PIT. If this were the case it would again imply that the ANT was mediating performance through the regulation of S-O or S-O-R relations, rather than the R-O relation, as discussed previously. In order to separate these possibilities it would have been necessary to show that the wheel turn was governed by its contingency with the footshock avoidance, *independent* of the S-O contingency. For example, Grindley (1932) showed that Guinea pigs who had learned to turn their head to the left or right every time a buzzer sounded to gain a carrot reward, would readily reverse the direction of head turning when the instrumental contingency was reversed but the S-O relation between the buzzer and carrot remained constant. Likewise if Gabriel et al. (1977, 1983, 1989) had shown that animals that had initially learned to turn the wheel in one direction to avoid shock and then learned to turn it in the opposite direction to avoid shock, independent of the continuing tone-shock contingency, this would suggest that the response was governed by the R-O, not the S-O contingency. Therefore, although elegant and among the first to assign a psychological function to a thalamic nucleus outside of general physiological functions, the research by Gabriel and colleagues leaves open the question of which type of relation governed behavior in these tasks and therefore which of these processes is regulated by the ANT.

A subsequent study by Corbit et al. (2003) specifically examined whether the ANT is required for the learning and expression of R-O relations. All of the behavioral tasks employed by Corbit et al. (2003) occurred in free-operant chambers of the kind described by Skinner (1932) meaning that no discrete stimuli were presented and the animal was free to emit (or omit) the behavior at any time in accordance with its expectation of receiving an outcome. Their first experiment employed the devaluation procedure described previously. Rats with sham lesions or ANT lesions were trained to make two instrumental responses (left and right lever presses) for two distinct outcomes (pellets and sucrose). Subsequently, one of these outcomes was fed to satiety to reduce its value, and rats were tested for their choice between levers in extinction. Both Sham control rats and rats with ANT lesions were able to preferentially choose the lever that had been associated with the non-prefed outcome during training (Figure 1C). As previously discussed, because no outcomes were delivered on test this result suggests that the performance of both the Sham and ANT lesioned rats relied upon the ability to recall the specific R-O contingencies. In their second experiment, Corbit et al. (2003) examined the effect of lesioning the ANT on the rats’ sensitivity to degradation of the instrumental contingency. For this task rats were exposed to the same R-O contingencies trained in Experiment 1, but one of the outcomes was also delivered in a manner that was not contingent on its associated lever press action. Both ANT lesioned rats and Sham controls showed evidence of degradation and reduced their responding on the lever earning an outcome that was also being delivered in a manner that was not contingent on lever pressing (i.e., degraded lever), whilst maintaining their response rate on the lever that continued to contingently earn an outcome (i.e., nondegraded lever: Figure 1D). This pattern was observed both during training and on a 10 min choice extinction test. Together, these experiments demonstrate that, in free-operant conditions, the ANT does not play a critical role in the acquisition and/or expression of instrumental actions. Taken together with the body of work presented by Gabriel and colleagues, these results suggest that the role of the ANT is more consistent with the regulation of Pavlovian processes or the Pavlovian control of instrumental behavior, particularly in aversive avoidance tasks. This conclusion is bolstered by findings of c-Fos related activity in the anterior thalamus after fear conditioning (Conejo et al., 2007) and the fact that lesions of the anterior thalamus cause deficits in the ability of rats to use S-O relations to escape from a water maze (Warburton and Aggleton, 1999).

## Mediodorsal thalamus

**Figure 2:**
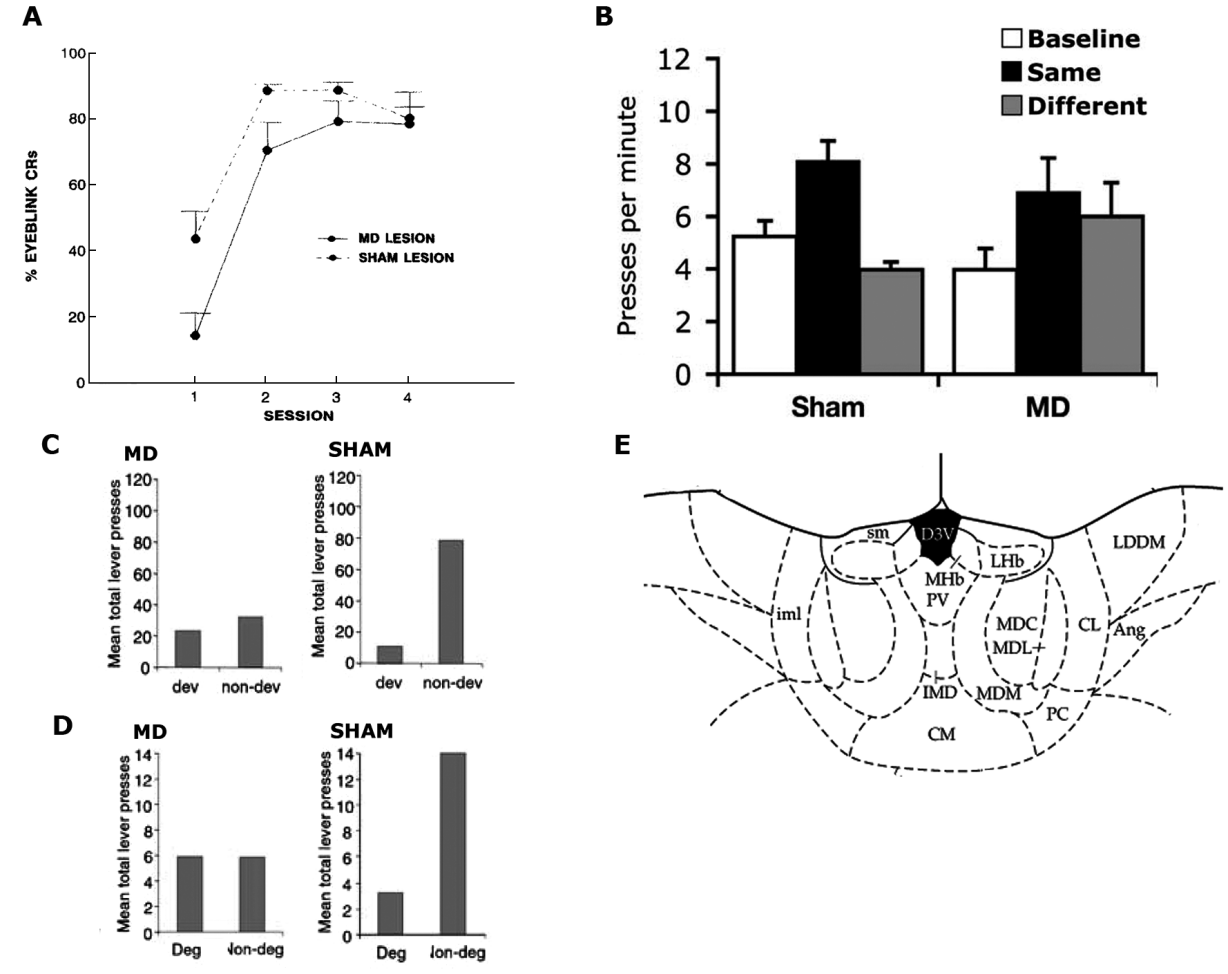
**(A)** Reproduced from Buchanan (1994). Mean ± SEM percentage conditioned eyeblink responses in MD and sham-lesioned animals. **(B)** Reproduced from Ostlund and Balleine (2008). Mean lever presses per min (± SEM) during the precue period (baseline), the cue that signalled the same outcome as the action (same) and the cue that signalled the outcome paired with the other action (different) in Sham and MD lesioned animals. **(C,D)** Reproduced from Corbit et al. (2003), examines sham and MD-lesioned animals. **(C)** Mean total lever-press responses per min for outcome devaluation test. **(D)** Mean total lever-press responses for the contingency degradation test. **(E)** Reproduced from Paxinos and Watson (1998). Schematic showing MD at A/P: −3.6 from Bregma.

A second thalamic candidate that has been examined within the literature for its role in the regulation of instrumental behaviors is the MD (see Figure 2E). As mentioned above, in some of the experiments conducted by Gabriel et al. (1989) and Smith et al. (2002) the ANT was not the only thalamic target of some of their manipulations, as the MD was also targeted some of the time. Although the pattern of results seemed to suggest a greater deficit when the ANT and MD were both targeted than when the MD was targeted alone (Gabriel et al., 1989), rabbits with lesions of the MD alone did show some deficit relative to controls. However, because these tasks confound Pavlovian and instrumental processes, there is some difficulty extracting information about the involvement of the MD in regulating instrumental behavior from these results.

Another early attempt at examining the role of MD in instrumental conditioning also involved rabbits with MD lesions but examined performance in an eyeblink avoidance conditioning task (Buchanan, 1994). During this task, rabbits were required to perform an eyeblink response during a tone presentation to avoid a shock US delivered around the eye. MD lesions impaired acquisition but not asymptotic performance (see Figure 2A), leading the author to conclude that the MD influenced responding through its role in a general process of attention or arousal rather than the acquisition of the instrumental contingency. It must again be considered, however, whether there is any evidence that the eyeblink response required for this task was instrumental. Like the aversive avoidance task, this task confounds the Pavlovian and instrumental relationships as the animal’s eyeblink response only achieves avoidance of the shock US when it occurs in the presence of the tone S+. Eyeblink responses that occur in the absence of the S+ bear no relation with the outcome such that, even if we accept that an eyeblink can be performed instrumentally, here it is controlled by the S+ in a manner that could be either S-O-R (implying no R-O control) or hierarchical S-(R-O). Thus, in the absence of any test to distinguish between these possibilities, the general depression in responding observed here in rabbits with MD lesions might reflect a general performance deficit but does not speak to the role of the MD in governing instrumental behaviors.

In the same series of experiments they used to examine the ANT, Corbit et al. (2003) provided an assessment of the MD’s role in regulating R-O relations. In marked contrast to the effects of ANT lesions, however, MD lesions did affect responding during outcome devaluation when tested in extinction (see Figure 2C). Importantly, when the outcomes were delivered on test, rats with MD lesions were able to choose the lever associated with the non-prefed outcome during training demonstrating they were able to discriminate between the two levers as well as encode the reduced value of the prefed food. This experiment also showed that the deficit observed in the extinction test could not be due to MD lesions affecting some kind of general process such as arousal or voluntary body movement, as such processes should have been equally affected during the rewarded test. A second experiment demonstrated that, again unlike ANT lesioned rats and controls, MD lesioned rats were not sensitive to selective reduction of the R-O contingency and responded similarly on both levers after degradation. This was observed both during training and test (see Figure 2D). Together these results clearly demonstrate a role for the MD in instrumental behavior. Moreover, they specifically suggest a role for the intact MD in regulating the acquisition of goal-directed instrumental behavior.

Ostlund and Balleine (2008) later re-examined the role of the MD in regulating instrumental performance. They again examined the effects of MD lesions but in this instance the lesions were performed after instrumental training. These post-training lesions produced a very different effect; although pre-training lesions abolished outcome devaluation it was unaffected by post-training lesions, suggesting that the MD plays a role in the acquisition of goal-directed behaviors but not their expression. This finding suggests that the MD might play a role similar to that of the prelimbic cortex (PL) which has been similarly found to mediate the acquisition but not expression of goal-directed behavior (Ostlund and Balleine, 2005), but differentiates it from the posterior dorsomedial striatum (pDMS) which has been shown to be mediate both acquisition and expression (Yin et al., 2005a,b).

In a second experiment, Ostlund and Balleine (2008) assessed PIT. In the Pavlovian training stage a tone stimulus was paired with pellets or sucrose and a white noise stimulus was paired with the alternate outcome. After eight days rats began the instrumental phase in which the left lever was paired with pellets or sucrose and the right lever paired with the other outcome. On test, the Pavlovian stimuli were presented while the rats were allowed to press both levers in the absence of outcome delivery. As is typically found, the Pavlovian cues biased performance towards the lever delivering the outcome predicted by the stimuli despite the rats never previously experiencing the stimuli and levers in the same session (Figure 2B). Rats with post-training MD lesions were unable to perform this task and pressed both levers equally during stimulus presentations. This result suggests that the MD not only governs reward guided actions but also stimulus guided actions, a result that offers some explanation as to why MD lesions, that impair goal-directed performance in the absence of explicit Pavlovian cues (Corbit et al., 2003), also impaired performance in aversive avoidance tasks potentially governed by Pavlovian processes (Buchanan, 1994). In contrast, however, it appears that when the outcome, rather than a predictive stimulus, is used as cue, MD lesions leave performance intact. That is, Ostlund and Balleine (2008) found that, when a pellet or sucrose outcome was delivered to the magazine after a period of extinction, responding was selectively reinstated on the lever associated with that outcome during training and to a similar degree in both the control rats and rats with MD lesions. This effect differs from transfer, however, in that the governing relation is not between the stimulus and outcome but between the stimulus and response (in which the outcome functions as the stimulus).

The last experiment in the series conducted by Ostlund and Balleine (2008) examined whether MD lesions affected performance during a Pavlovian contingency degradation task that employs alterations in the predictive S-O relationship. For this task the rats continued to receive the same S-O pairings received in previous Pavlovian training, but one of these outcomes was also delivered unpaired with any stimuli. This served to degrade the contingency between the stimulus and that outcome as Sham rats selectively reduced time spent in the magazine during presentations of that stimulus. Rats with MD lesions, on the other hand, reduced responding to both stimuli, suggestive of a specific deficit in the encoding of S-O relations.

Taken together, these experiments demonstrate the complex nature of the MD’s role in instrumental behavior. On the one hand, pre-training MD lesions impaired the acquisition of R-O contingencies and the selective degradation of one of these contingencies, suggesting that an intact MD is crucial for the acquisition of instrumental behaviors guided by R-O relations. On the other hand post-training MD lesions left outcome devaluation intact whilst impairing Pavlovian-to-Instrumental transfer and Pavlovian contingency degradation. Perhaps the simplest explanation for the multiple functions of the MD lies in the diverse connections it maintains with the frontal cortex. Connections between the MD and the prelimbic prefrontal cortex of the rat are, at least anatomically, the best studied (Groenewegen, 1988; Kuroda et al., 1993), but the MD also maintains strong connections with the orbitofrontal cortex (OFC) particularly its lateral regions (Krettek and Price, 1977). Recent studies have found that, whereas the prelimbic area is critical for the acquisition of goal-directed instrumental actions, it plays little if any role in appetitive Pavlovian conditioning or in the influence of Pavlovian cues on instrumental performance (Corbit and Balleine, 2003). In contrast lesions of lateral OFC, whilst sparing instrumental acquisition, abolish the outcome specificity of Pavlovian S-O relations (Schoenbaum and Roesch, 2005; Ostlund and Balleine, 2007) together with outcome-specific Pavlovian instrumental transfer (Ostlund and Balleine, 2007; Balleine et al., 2011). Hence, it seems likely that the diverse functions of the MD reflect the important role it plays in the distinct functions of the frontal cortical regions to which it projects.

## Prelimbic-mediodorsal thalamus interactions: the effect of disconnecting the thalamo-cortical pathway on goal-directed instrumental actions

The heavy interconnectedness of the MD and PL (Groenewegen, 1988) and their similar role in the acquisition of goal-directed instrumental actions led us to hypothesize that the encoding of the R-O contingency depends on the PL-MD pathway. In particular, we predicted that a functional disconnection of PL and MD would abolish goal-directed behavior. By contrast, we predicted that there would be no deficit in rats that received a functional PL/MD disconnection in outcome-induced reinstatement performance that tests the acquisition of O-R rather than R-O contingencies, particularly as bilateral PL lesions leave reinstatement unaffected (Ostlund and Balleine, 2005) as do MD lesions (Ostlund and Balleine, 2008).

Not only are the connections between PL and MD large and reciprocal, the PL projects to the MD in both the ipsilateral and contralateral hemispheres (Buchanan, 1994). Therefore, a traditional lesion disconnection study, in which rats might receive PL and MD lesions in contralateral hemispheres, should not be sufficient to anatomically or functionally disconnect these structures. That is, although it would disconnect these structures ipsilaterally, it would leave the contralateral PL-MD projections intact. For this reason, there have been few studies directly examining of the effect of disconnecting the PL with various sub-cortical structures. One notable attempt was that of Coutureau et al. (2009) who contralaterally lesioned the PL and basolateral amygdala (BLA) and found that although bilateral lesions of either structure abolished goal-directed responding their disconnection did not. It is possible that this failure to find an effect was because these structures communicated via the remaining contralateral projections, despite the authors arguing that these cross-connections are only weak. If it was not due to these connections then this finding is illustrative of the fact that disconnections do not always have the same behavioral consequences as bilateral lesions of those structures, demonstrating the necessity of testing disconnections in spite of the lesion data. In contrast to the PL-BLA pathway, PL-MD contralateral projections are substantial (Negyessy et al., 1998), so to ensure a full functional disconnection of PL and MD structures in the current study we included an electrolytic lesion of corpus callosum (CC) to specifically sever the contralateral projections. All experimental and surgical procedures were approved by the Animal Ethics Committee at the University of Sydney, and are in accordance with the guidelines set out by the American Psychological Relation for the treatment of animals in research.

**Figure 3:**
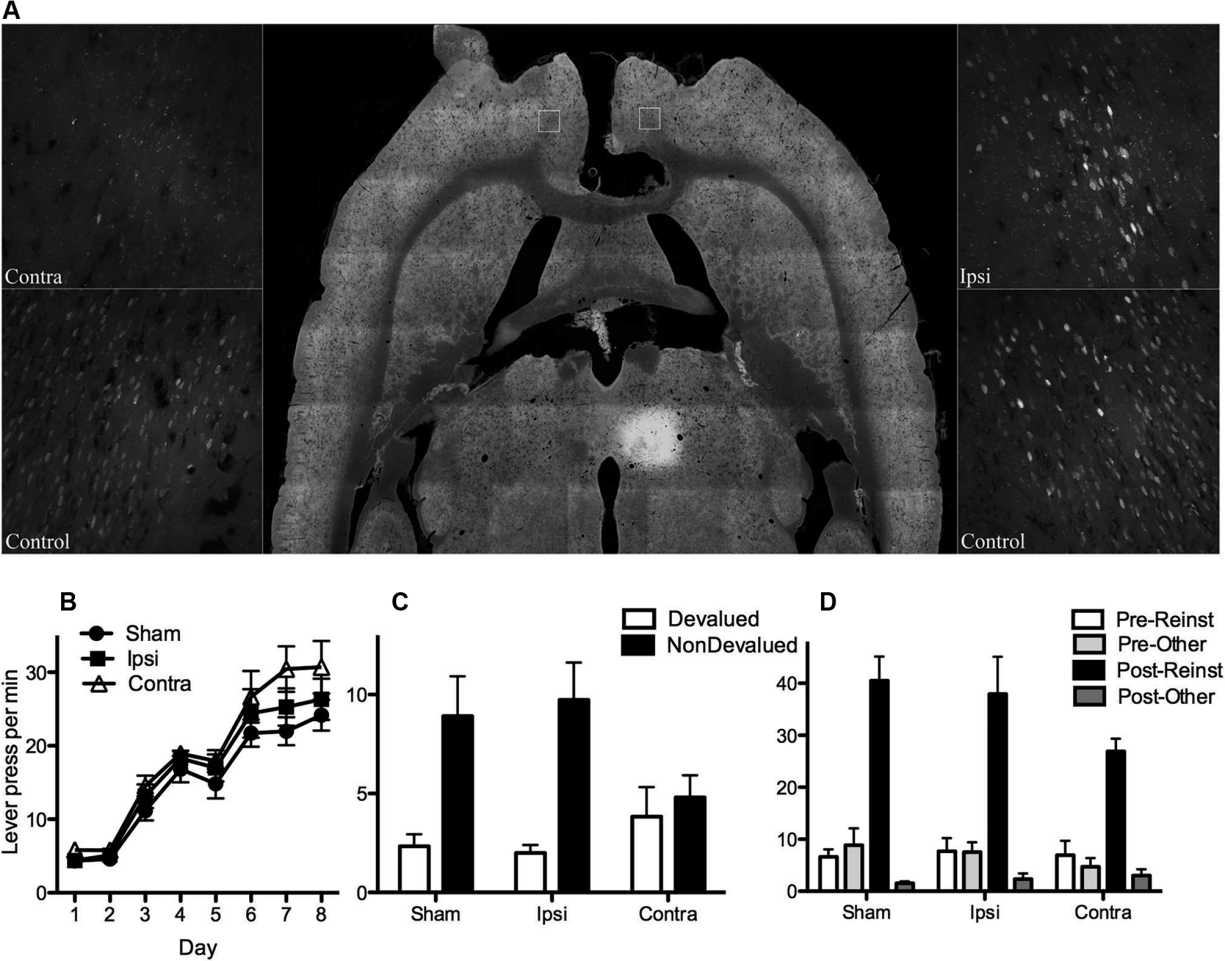
**(A)** Shows the extent of fluorogold labeling in prelimbic cortex (PL) after receiving an injection of retrograde tracer FG into MD and either electrolytic (Contra and Ipsi) or sham (control) lesions of corpus callosum (CC). Horizontal section (middle panel) shows injection site in MD as well as CC lesion. CC lesions did not affect ipsilateral projections (no difference in labeling in Ipsi and control, right panel) but were effective in disconnecting contralateral projections (very little labeling in Contra relative to control, left panel). **(B)** Mean (± SEM) lever presses per min for the control groups (Groups Ipsi and Sham) and Group Contra that suffered a functional PL-MD disconnection (i.e., CC lesion plus contralateral N-methyl-D-aspartate (NMDA)-induced lesions of PL and MD). For all statistical analyses Group Sham and Ipsi did not differ on any measure (all *Fs* < 1) and therefore were averaged across for further analysis. All rats linearly acquired lever press responding, *F*(1, 19) = 226.00, *p* = .00, and groups did not differ on acquisition, *F*(1, 19) = 2.194, *p* = .16. **(C)** Mean (± SEM) lever press responding per min during outcome devaluation testing. Groups did not differ in overall responding, *F*(1, 19) = 1.19, *p* = .29, but there was a main effect of devaluation (averaged over group), *F*(1, 19) = 18.54, *p* = .00. There was a significant interaction, *F*(1, 19) = 5.79, *p* = .026, suggesting that both the control groups responded selectively on the nondevalued lever relative to the devalued lever (simple effects: Group Sham, *F*(1, 19) = 10.08, *p* = .008, Group Ipsi, *F*(1, 19) = 14.76, *p* = .001) but that Group Contra responded equally on both levers (simple effect: *F*(1, 19) = .24, *p* = .63). **(D)** Mean (± SEM) lever press responding per min during outcome-induced reinstatement testing. There was a main effect of reinstatement, *F*(1, 19) = 105.38, but no group x reinstatement interaction, *F*(1, 19) = 3.88, *p* = .065. Although this interaction might be considered marginal, simple effects show that rats in each group pressed the reinstated lever more than the other lever on test, Group Sham, *F*(1, 19) = 54.31, *p* = .00, Group Ipsi, *F*(1, 19) = 39.6, *p* = .00, and Group Contra, *F*(1, 19) = 17.81, *p* = .00.

First we demonstrated the efficacy of lesioning the CC in severing these contralateral PL-MD projections. After this lesion had been made the retrograde tracer fluorogold (FG) was injected unilaterally into the MD of five Long-Evans rats. Brains were later examined for the extent of labeling in the PL in both hemispheres: that which was ipsilateral and that which was contralateral to FG injection. From Figure 3A it is clear that almost no FG labeling was observed in the PL contralateral to the MD injection site relative to that observed in a control rat that had no CC lesion. This suggests that the CC lesion was successful in severing contralateral projections between these structures. By contrast, it is also clear from this figure that ipsilateral projections were unaffected by the CC lesions: labeling in the hemisphere ipsilateral to the injection site looked similar in both lesioned rats and unlesioned control rats.

Once the efficacy of the CC lesion in severing these projections had been determined, 30 experimentally naïve Long-Evans rats received CC lesions combined with sham or excitotoxic PL and MD lesions with the aim of examining the effect of disconnecting these structures on outcome devaluation, and outcome-induced reinstatement. Eight of these had misplaced lesions or damage that extended beyond the CC and thus were excluded from the analysis. Twenty two rats were then used for analysis. There were three groups: Group Sham (*n* = 8), Group Ipsi (*n* = 7), and Group Contra (*n* = 7). Each rat in each group received a CC lesion. Rats in Group Ipsi received additional excitotoxic lesions of PL and MD in the same (ipsilateral) hemisphere such that these structures were disconnected in that hemisphere but an intact PL-MD pathway remained in the opposite hemisphere. Rats in Group Contra received additional excitotoxic lesions in alternate hemispheres such that the PL-MD pathway was disconnected in both. Therefore Groups Ipsi and Contra differed only in the hemispheric location but not the overall amount of damage. Rats in Group Sham controlled for the effects of receiving a CC lesion with sham PL and MD lesions (in which the needle was inserted but no excitotoxin injected). Half of the sham lesions were given ipsilaterally and half were given contralaterally. In addition, the hemispheres in which damage occurred were counterbalanced within each group (i.e., left vs. right).

For the next eight days rats received instrumental training. For half of the rats in each group the left lever earned pellets and the right lever earned sucrose. The remaining rats were trained on the opposite R-O contingencies. Acquisition of lever press responding is shown in Figure 3B. From this figure it is clear that all groups acquired lever press responding and that the groups did not differ (see Figure for statistical analysis). Subsequent to lever press training rats were tested for knowledge of these contingencies. There were two tests, one with pellets and one with sucrose (counterbalanced). Prior to each test rats received free access to either outcome to specifically satiate them on this outcome thereby reducing its value relative to the non-prefed outcome (cf. Balleine and Dickinson, 1998). As a result rats in the Sham and Ipsi control groups were expected to choose the lever that had been associated with the nondevalued outcome during training. As described previously, testing was conducted in extinction such that rats were required to rely on their knowledge of the R-O contingency to choose the nondevalued over the devalued lever. Test performance, averaged over the two tests, is shown in Figure 3C. As expected, rats in Groups Sham and Ipsi demonstrated evidence of having acquired the R-O contingencies (nondevalued > devalued) whereas rats in Group Contra did not (nondevalued = devalued, see figure caption for statistical analysis). This result suggests that the functional disconnection of PL and MD mimicked that of bilateral lesions of either structure; i.e., rats with functional disconnection of the PL-MD pathway demonstrated a decrement in goal-directed learning relative to Sham and Ipsi controls.

Finally, we examined whether rats in each group would selectively reinstate responding on the lever that had been associated with a particular outcome during training. Specifically, after 15 min of extinction on both levers, rats received four reinstatement trials separated by 7 min of extinction in which a pellet or sucrose outcome was freely delivered and responding recorded for the next 2 min. Outcomes were delivered in the order: pellets, sucrose, sucrose, pellets. It was expected that pellet delivery would reinstate responding on the lever that had earned pellets during training, and similarly sucrose delivery would reinstate responding on the sucrose lever. Results are shown in Figure 3D. It is clear from this Figure that all groups showed greater responding following outcome-delivery and that this increase in responding was selective for the reinstated lever (i.e., reinstated > other, see figure caption for statistical analysis). Although the bilateral lesions of PL and MD have no effect on outcome-induced reinstatement, it was important to demonstrate that the functional disconnection of these structures left reinstatement performance intact. This is because it rules out several potential explanations of the impairment in the outcome devaluation test, including a simple deficit in discriminating between levers and outcomes.

Together, these results show that disconnecting the PL-MD pathway creates a deficit in outcome devaluation performance whilst leaving outcome-induced reinstatement intact. The deficit observed during outcome-devaluation suggests that the MD does rely on inputs from the PL (or vice versa) for accurate performance in this task. Intact reinstatement suggests that this deficit was not a result of impaired discrimination, and the fact that there was no difference in lever-press acquisition suggests that the deficit in outcome devaluation performance cannot have resulted from a lack of opportunity to learn the R-O contingencies. Rather, the pattern of results suggests that this group suffered a specific deficit in using R-O contingencies to guide action selection such that they pressed both levers equally on test.

It is worth pointing out here that the success of the novel surgical technique involving electrolytically lesioning the CC in inducing a full functional disconnection of the PL and MD, as evidenced by the lack of FG labeling observed in the PL contralateral to the MD injection site as well as the behavioral deficit observed, could have wide-ranging implications. In particular, researchers who might have previously wished to examine the effect of disconnecting prefrontal cortical (and other cortical) structures from subcortical structures with which they share ipsilateral and contralateral connections now have a potentially viable technique with which to do so. For example, Hunt and Aggleton (1998) found that lesions of both regions produce similar deficits in shifting response rules during a radial arm maze task. Likewise, Balleine and Dickinson (1998) found that PL lesions, like MD lesions described above, reduced responding non-selectively on both levers during a contingency degradation task. And, similarly, Ostlund and Balleine (2007, 2008) found similar effects on Pavlovian instrumental transfer induced by lesions of the MD and lateral OFC. Thus, given the similarity of these deficits produced by lesions of the MD and frontal cortical structures in behavioral tasks other than those reported here, it might be hypothesized that functionally disconnecting these structures will produce a similar deficit. Until now it has not been possible to explore such questions. Therefore, the surgical procedure described in the current study provides an exciting prospect for the study of these, and other potential functions of the thalamo-cortical pathway.

## The parafascicular thalamic nucleus

**Figure 4:**
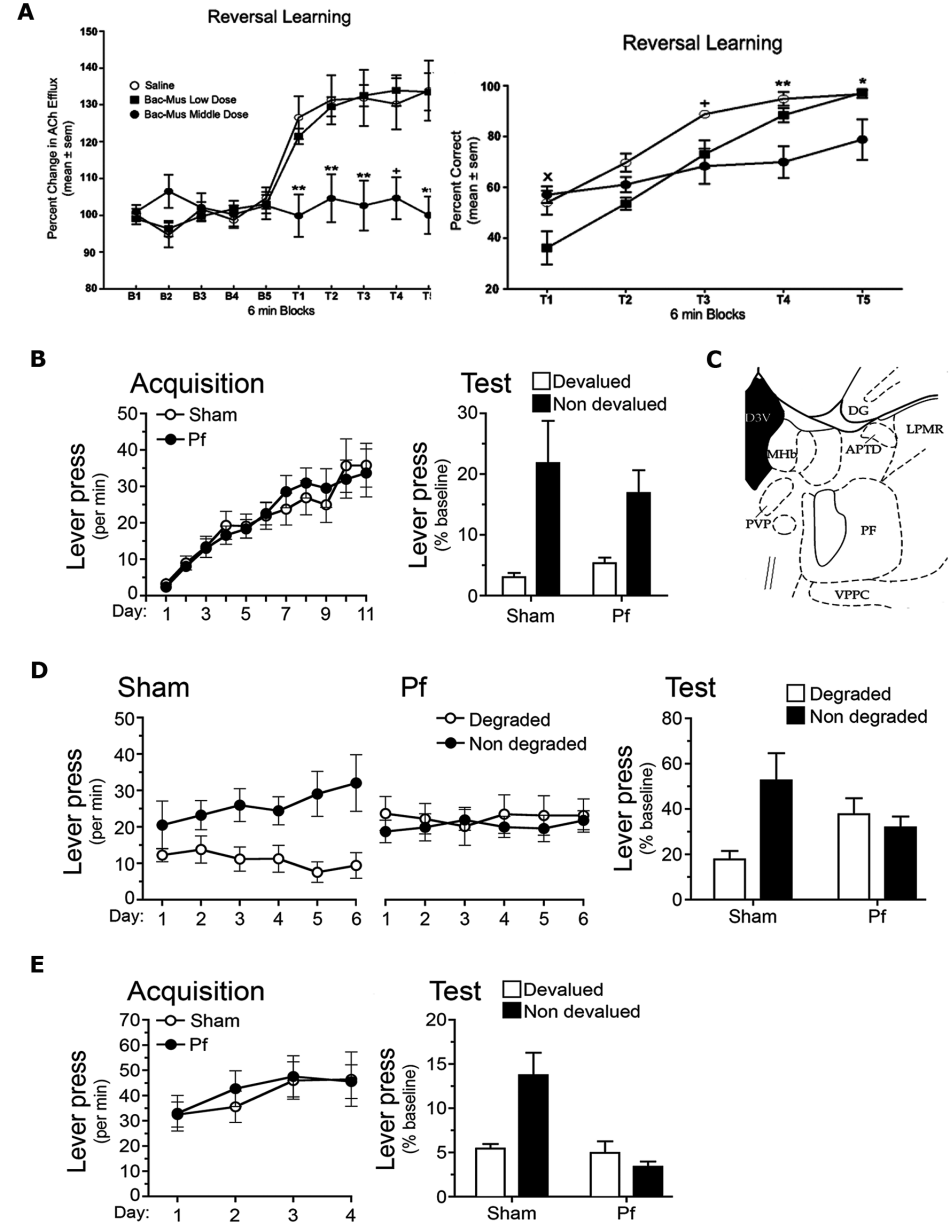
**(A)** Reproduced from Brown et al. (2010). Acetylcholine (Ach) efflux in anterior dorsomedial striatum (aDMS; left panel) and behavioral performance (right panel) during 6 min blocks during the reversal learning phase of a T maze task (T1–T5). Left panel: the middle dose of GABA_A_ agonist Baclofen-Muscimol (Bac-Mus) infused into the parafascicular thalamus (PF) was sufficient to reduce Ach efflux in the aDMS during reversal learning. Right panel: infusion of the low dose of Bac-Mus significantly reduced reversal learning performance relative to saline-infused controls at T1. The Middle dose reduced performance at T3, T4, and T5 relative to saline controls. **(B,D,E)** Reproduced from Bradfield and Balleine (2013), examines Sham and PF-lesioned animals. **(B)** Mean ± SEM responding per min during acquisition (left panel) and on an outcome devaluation test (right panel) of initial R-O contingencies. **(C)** Reproduced from Paxinos and Watson (1998). Schematic showing PF at A/P: −4.16 from Bregma. **(D)** Mean ± SEM responding per min during contingency degradation training (left and middle panels) and on the extinction test (right panel). **(E)** Mean ± SEM responding per min during acquisition (left panel) and test (right panel) of reversed R-O contingencies.

The final region we consider for its role in instrumental behavior was that of the parafascicular thalamic nucleus (PF; see Figure 4C). The PF was one of the first thalamic regions to be assessed for its role in instrumental behavior. Delacour (1969) found that lesioning the PF did not affect learning during a passive avoidance or one-way avoidance task. Of some interest, however, were the findings of the second experiment showing that although PF lesioned rats were unimpaired relative to controls in learning to cross from shocked compartment A to the non-shocked compartment B, they did show a deficit when the shocked compartments were switched and rats had to learn to cross in the other direction (i.e., from B to A). This inability to reverse the previously learned contingency suggests a potential deficit in flexible performance. Unfortunately, in this instance it is not possible to separate the inflexibility of PF lesioned rats in learning a new Pavlovian relation (i.e., “stimulus” compartment A → “outcome” avoid shock) from inflexibility in learning a new instrumental action (i.e., “response” cross to A → “outcome” avoid shock).

The suggestion that the PF might regulate the flexibility of instrumental behavior was re-visited by Minamimoto et al. (2009) using primates as subjects. They recorded the responses of long latency facilitation (LLF) neurons in the centro-median parafascicular nuclear complex (CM-PF; the primate homologue of the PF) of two monkeys during a GO-NOGO task. This task requires monkeys to either respond (“GO”) or withhold responding (“NOGO”) to particular stimuli to receive a large or small water reward. LLF neurons in the CM-PF showed an interesting pattern of responding during the trial blocks when the GO response was paired with the large reward and the NOGO response paired with the small reward. Specifically, after several NOGO trials the likelihood of a GO trial increased, in parallel with the likely increase in the monkey’s expectation of a GO response. When the NOGO stimulus was then unexpectedly presented LLF activity increased, but only when a NOGO response was produced. When the response was not produced LLF activity remained weak or silent, indicating that the presentation of an unexpected stimulus alone was not sufficient for this increase in LLF activity. The authors interpreted this as showing that CM-PF LLF neurons drive a kind of “rebias” process that occurs when the animal expects to produce one response but quickly changes to another. This, like Delacour (1969) study appears to point to a role for CM-PF in flexible responding, a requirement of instrumental conditioning. Again, however, it is difficult to make a solid conclusion about PF regulation of instrumental conditioning because the response measured in this task was stimulus-dependent, therefore already somewhat inflexible. Further, the activity of LLF neurons was purely correlative such that interpreting the activity of LLF neurons to be reflective the monkey’s expectations was somewhat speculative.

More recently, Brown et al. (2010) conducted a series of experiments that they also interpreted as showing that the PF mediates behavioral flexibility, and does so by influencing acetylcholine (ACh) levels in the aDMS, with which it has large and specific connections. These experiments employed a T maze task in which rats were placed into the stem arm and learned to travel to one of the choice arms to retrieve a piece of cereal. Rats were trained to criterion (10 consecutive correct trials) in this phase of the task and then trained to enter the opposite arm (“reversal phase”) the following day; i.e., the previously non-reinforced arm became the reinforced arm. Half of the rats received intra-PF infusions of baclofen-muscimol (Bac-Mus) prior to the initial acquisition and half received saline. PF inactivation did not affect initial acquisition because all rats took the same amount of time to reach criterion. However, rats that received Bac-Mus infusions prior to the reversal phase did take longer than saline-infused animals to reach criterion. In a separate experiment Brown et al. (2010) found that reversal learning of this kind increased ACh efflux in the DMS during reversal training, and that PF inactivation (using Bac-Mus) prevented this increase (see Figure 4A). They concluded that this increase in ACh efflux depended on PF inputs and reflected a facilitation in altering choice patterns and thus behavioral flexibility.

Although it is possible that this efflux in ACh did indeed reflect a facilitation of flexibility, this is not the only interpretation of these results. This is because behavioral flexibility implies an exclusively instrumental process, and it is particularly difficult to disentangle the Pavlovian and instrumental processes that might be employed during performance in T-maze tasks such as the one employed by Brown et al. (2010) (Dickinson, 1994; Yin and Knowlton, 2002; Yin et al., 2008). First, without a test session in which the outcome is absent it is not possible to conclude whether performance is governed by R-O contingency knowledge or whether the animal simply becomes better at detecting the presence of food over time. Second, without an evaluation of whether altering the value of the cereal also altered performance in the task there is no necessary demonstration that the cereal behaves as a “goal” of behavior. Brown et al. (2010) did not include either of these tests making it equally as likely that performance on this task was governed by S-O relations between maze cues and the cereal outcome. This conclusion holds even in spite of the maze being turned between trials to minimize the consistency of external cues; making extramaze cues ambiguous could have forced the rats to rely on intramaze cues (e.g., walls, floor). Superficially it might appear that the reversal learning assessed by Brown et al. (2010) addresses this problem by demonstrating that rats are able to flexibly alter responding in the manner required of an instrumental response. However, it is necessary to show that this occurs when the S-O relation is kept constant. In T-maze reversal learning, if the animal first learns to turn right on reversal, then the stimuli associated with turning left (i.e., the arm of the “T” that leads left) are no longer associated with reward, leading to extinction of that S-O relation. Instead, the animal forms a new S-O relation between the stimuli associated with turning right and the reward. In other words, it is not possible to show that animals can alter responding whilst keeping S-O relations consistent using a T-maze task.

A final point to be made about these experiments (Brown et al., 2010) is that the microdialysis probe placed in the DMS to measure ACh efflux was aimed aDMS whereas prior research has found that it is specifically the pDMS that mediates goal-directed instrumental conditioning (e.g., Yin et al., 2005a,b). Specifically, Yin et al. (2005a,b) used outcome devaluation and contingency degradation procedures similar to those described here and found that although the pDMS is critically involved in both the acquisition and expression of goal-directed behaviors, manipulations of aDMS had no effect. Furthermore, a separate study has suggested that, rather than governing instrumental behavior, the aDMS regulates the Pavlovian control of behavior (Corbit et al., 2007). On this basis, we suggest that the alterations in the behavior of PF-inactivated rats in the study by Brown et al. (2010) and the concomitant increase in ACh in aDMS, reflects the role of the PF-aDMS pathway in facilitating learning about alterations S-O rather than R-O relations.

More recently we have investigated whether the PF mediates behavioral flexibility via its afferents to the pDMS using unambiguous manipulations of the R-O contingency (Bradfield et al., 2013). In particular, we examined the role of PF-controlled tonically active pDMS cholinergic interneurons (CINs) in the interlacing of new and existing R-O contingencies. We first examined the effects of bilateral PF lesions on the same outcome devaluation and contingency degradation procedures outlined previously. Although PF lesions did not affect outcome devaluation (Figure 4B) and therefore did not interfere with the initial acquisition of goal-directed behaviors, PF lesioned rats did show a deficit relative to controls during contingency degradation (Figure 4D). Specifically, while the Sham control rats selectively reduced their responding on the degraded lever during training and test, PF lesioned controls continued to press both levers equally. One interpretation of this result is that once PF lesioned rats had learned the initial R-O contingency, the rats were unable to alter (reduce) their responding when the contingency changed. Because other interpretations of this result are possible, a third experiment was conducted to test this hypothesis. For this experiment the contingencies learned in the initial training phase were reversed, for example, if the left lever was previously paired with pellets it was now paired with sucrose, and if the right lever was previously paired with sucrose it was now paired with pellets. Sham rats demonstrated devaluation performance in line with the reversed contingencies because when they were prefed one of the outcomes to satiety and then tested in extinction, they preferentially chose the lever associated with the nondevalued outcome. PF lesioned rats, on the other hand, chose both levers equally (Figure 4E). This deficit was not limited to performance in a devaluation/extinction test. When the rats were treated to 15 min of extinction and then delivered two single pellet and two single sucrose presentations (separated by 7 min ITIs), Sham rats selectively reinstated responding on the lever associated with the relevant outcome according to the reversed contingencies, whereas PF lesioned rats pressed both levers equally. Together with the observed deficit in contingency degradation, this result suggests that an intact PF is necessary for true behavioral flexibility.

These behavioral tasks were repeated in later experiments to test the effects of functionally disconnecting the PF-pDMS pathway. Prior to these experiments we had injected the retrograde tracer FG into the pDMS and evaluated labeling in the PF. This confirmed that the PF does project to the pDMS and that the pathway is entirely lateralised. Rats were then administered either Sham, ipsilateral, or contralateral, PF/pDMS lesions. Rats with ipsilateral PF-pDMS lesions (group Ipsi) retained an intact PF-pDMS pathway in the opposing hemisphere, whereas contralateral PF and pDMS lesions (group Contra) ensured rats had no intact pathway in either hemisphere. Thus both the Sham and Ipsi groups controlled for the behavior of group Contra. Rats in this group (group Contra) showed the same pattern of results as bilaterally PF lesioned rats. That is, they showed intact initial acquisition of R-O contingencies, but impaired contingency degradation and acquisition of the reversed R-O contingencies. In contrast Sham and Ipsi rats showed intact performance in all tasks. After the rats were sacrificed their brains were sectioned and examined for examined p-Ser^240-244^-S6rp intensity in cholinacetyltransferase (ChAT) immunoreactive neurons in the non-lesioned pDMS. p-S6rp was recently shown to reflect the activation levels of CINs particularly well (Bertran-Gonzalez et al., 2012). Analysis of the results showed that p-S6rp intensity was significantly reduced in Group Contra relative to Groups Ipsi and Sham, reflecting the reduced inputs from the lesioned PF in this group relative to the other two groups. A separate experiment using patch-clamp electrophysiology showed that removing PF inputs to the pDMS by lesioning the PF reduced the frequency of action potentials in pDMS CINs. A final experiment examined the effect of compromised CINs function on behavior. For this experiment all rats had a unilateral PF lesion, and then received an infusion of either saline or the M2/M4 muscarinic receptor agonist Oxotremorine-S (Oxo-S) into the contralateral pDMS prior to learning reversed R-O contingencies. On test, saline-infused rats demonstrated evidence of having learned the reversed contingencies (nondevalued > devalued) but Oxo-S-infused rats did not (nondevalued = devalued). Together, these results suggest that the activation of CINs in the pDMS is reliant on PF inputs, and is necessary for the flexible responding in the face of altered R-O contingencies.

Given that PF also innervates the aDMS, and that Brown et al. (2010) found that ACh increases in a manner that is dependent on PF inputs during a behavioral task, we also considered the effect of disconnecting the aDMS-PF pathway. Although aDMS lesions are known to have no effect on the initial acquisition of R-O contingencies, nor their degradation (Yin et al., 2005b), the effect of such lesions on learning reversed contingencies was unknown. Using the same asymmetrical lesion design, but substituting aDMS for pDMS lesions, we found that disconnecting this pathway left the acquisition of both initial R-O contingencies and their reversal intact, suggesting that any increases in aDMS ACh that are observed during a behavioral task either are functionally irrelevant for the interlacing of new and existing R-O contingencies, or that similar increases do not occur in tasks requiring flexibility of R-O contingencies.

This role of the PF (via inputs to the pDMS) differs from that of the MD in instrumental behavior in that the former is necessary for interlacing new and existing R-O contingencies, whereas the latter is necessary for the initial acquisition of R-O contingencies. Thus both of these thalamic regions play different but vital roles, however, whereas an intact MD is critical for a naïve animal to carry out various tasks to achieve an outcome, an intact PF is critical for animals to continue to perform these tasks when environmental contingencies change. Any animal that lacks either function would be at a distinct disadvantage.

The results regarding the PF are also consistent with another critical function: the regulation of what recent computational views of instrumental conditioning have referred to as “state prediction errors”. State prediction errors differ from reward prediction errors, that regulate learning during both Pavlovian and Instrumental conditioning and for which the neural mechanisms have been reliably established (Schultz and Dickinson, 2000; Waelti et al., 2001; Steinberg et al., 2013). The idea of “state” prediction has arisen with the recent increase in popularity of computational models (e.g., Daw et al., 2005) that model aspects of instrumental conditioning. These types of models suggest that model-based goal-directed behavior is observed when an animal experiences a series of transitions from one state (akin to a “situation”) to another that ultimately results in the acquisition of a particular outcome. After encoding these state-to-state transitions the animal is then able to use its previous experiences to conduct a kind of forward search through the various states to ascertain whether their actions will lead to the acquisition of the outcome. State prediction errors are generated when the animal enters a state that is surprising given the probability with which they currently estimate their state-to-state transitions.

Experimentally, state and reward prediction errors tend to co-occur and are difficult to separate behaviorally (Schoenbaum et al., 2013). One experiment that does separate them, however, is the reversal of existing R-O contingencies. Upon entering the initial state during the reversal phase of the experiment, the animal expects that pressing the left lever (for example) will lead him to the state in which pellets are delivered to the magazine. When the animal is surprisingly transitioned into a different state in which sucrose is delivered instead, a large state prediction error is generated. If, however, it is assumed that rats value pellets and sucrose equally, then reward prediction error is zero because there is no discrepancy between the actual and expected reward. Therefore, the inability of rats with a compromised PF-pDMS pathway to accurately learn the reversed contingencies is consistent with an inability of these rats to effectively encode state prediction error. To be more specific, it is consistent with an inability to encode a reduction in contingency learning as a result of state prediction error. This is because the performance of PF-pDMS-compromised rats on this task was indiscriminate (i.e., they press equally on both levers at test, refer to figure). If these rats were incapable of encoding an increase in learning as a result of state prediction error, they should show no evidence of having learned the new contingencies (e.g., “left lever surprisingly leads to the state in which sucrose is delivered”) and show greater responding on the now-devalued lever than the nondevalued lever on test. If, however, these rats were specifically incapable of encoding a reduction in learning that resulted from state prediction error (e.g., “left lever no longer leads to the state in which pellets are delivered”) they would fail to unlearn the old contingencies whilst still learning about the new contingencies and their performance would be confused between the two on test. That is, they should show respond equally on the devalued and nondevalued levers, as observed.

Contingency degradation results also support this conclusion. PF-pDMS compromised rats, unlike controls, failed to reduce their responding on the degraded lever. State prediction error contributes to the reduction in learning about the degraded lever-outcome contingency during contingency degradation. Specifically, there is a state prediction error when an outcome that was previously paired only with lever press is also delivered outside of the lever press contingency. When the outcome was dependent on lever press alone, the animal learned that only pressing the lever in the initial state would transition them to the next state in which a pellet (for example) is delivered to the magazine. During contingency degradation they are surprisingly transitioned to this state without pressing the lever, generating a state prediction error. This state prediction error triggers an increase in learning (that favours learning about context-outcome relations) but also a reduction in learning about the contingency between performing the lever press in the initial state and entering the “food delivered” state. It is this reduction that leads to decreased responding on the degraded lever. Thus, the fact that the PF-pDMS compromised rats do not decrease responding on the degraded lever throughout training, is again consistent with an inability of those rats to process state prediction error in a manner that leads to a reduction in R-O contingency knowledge.

It is important to mention that, although broadly consistent with this view, Schoenbaum et al. (2013) have developed an alternative interpretation of these results. In a similar fashion to our interpretation (Bradfield et al., 2013), Schoenbaum et al. (2013) suggested that the PF-pDMS compromised rats primarily suffered a deficit in processing errors concerned with identity rather than reward. However, where we interpreted “states” in the manner assumed by model-based and model-free reinforcement learning models, Schoenbaum et al. (2013) offered an alternate but equally valid interpretation suggesting that the encoded states were more akin to a “context” or “latent cause”. On this account the state refers to the phase of training that the animal enters when it encounters an alteration in contingency, such as in contingency degradation (“state 2”) or reversal learning (“state 3”). It is Schoenbaum et al. (2013) suggestion that the PF-pDMS compromised animal suffers either a retrieval deficit such that multiple states are retrieved at one time causing confusion to the animal, or a state creation deficit in which the animal is incapable of forming a new state based on errors in identity prediction.

## Conclusion

Research regarding the role of various thalamic nuclei in instrumental behavior has increased in recent years. One of the earliest regions considered were the ANT. Although early indications appeared to suggest that ANT did indeed mediate instrumental behavior, careful examination of these tasks revealed that the learning processes governing behavior confound Pavlovian and instrumental processes. By contrast, when rats with ANT lesions were tested in free operant instrumental conditions they showed no deficits in a range of tasks (Corbit et al., 2003) effectively excluding this region as a candidate for the regulation of instrumental behavior as governed by R-O relations.

Another region that has received attention for its role in regulating instrumental behavior is the MD. Again, early indications suggested a possible role for this region but did not employ tasks that clearly separate Pavlovian and instrumental relations. In contrast to the ANT, however, MD lesions were later found to affect performance in several free operant behavioral tasks, highlighting a specific role for this region in the regulation of goal-directed instrumental behavior (Corbit et al., 2003). In addition, later research (Ostlund and Balleine, 2008) found that although the MD was important for the acquisition of goal-directed behavior, it was not important for its expression, as post-training MD lesions left goal-directed behavior intact. Finally, in the same study, it was found that an intact MD was important for the regulation of S-O as well as R-O contingencies, as MD lesions abolished PIT performance and Pavlovian contingency degradation.

Given that PL lesions regulate the acquisition but not expression of R-O contingencies in the same manner of MD lesions, we examined the effect of their disconnection in the current study. Because there are contralateral, as well as ipsilateral, connections between PL and MD, this required the adoption of a novel surgical technique that involved electrolytic lesions of the CC. Once the efficacy of this procedure in severing contralateral PL-MD connections had been established using the retrograde tracer FG, a functional disconnection of these structures was employed to examine the effect of this disconnection on various behavioral tasks. Specifically, all rats received Sham, ipsilateral or contralateral excitotoxic lesions of PL and MD in addition to a CC lesion. We found that outcome-induced reinstatement performance was intact in all groups, but that Group Contra showed a specific deficit in outcome devaluation testing. This suggests that the PL-MD pathway regulates learning of R-O contingencies in a manner that cannot be attributed to a deficit in discrimination or some other general process important to learning.

Finally, the role of the PF was examined, in particular for its role in flexible of instrumental behavior. Although several earlier studies implicated such a role for PF, again these tasks made it difficult to separate the influence of Pavlovian and instrumental processes. Our recent research by Bradfield et al. (2013) has shown, however, showed that the PF, via its control of the tonic activity of pDMS CINs, mediates the alterations in learning that occur when R-O contingencies change. This role is notably consistent with the possibility that PF-controlled pDMS CINs encode state prediction error, in particular when that error leads to reductions in contingency knowledge.

In summary, then, it is clear that there are multiple important and contrasting roles of various thalamic nuclei in the regulation of instrumental behavior. Given the wide connectivity of these nuclei with many striatal and cortical regions of interest, this is unsurprising. Future research will continue to uncover the specific role of these regions, particularly in the context of the complex interplay these regions enjoy with other structures in the brain.

## Conflict of interest statement

The authors declare that the research was conducted in the absence of any commercial or financial relationships that could be construed as a potential conflict of interest.
